# Arterial Stiffness, Assessed Using the Cardio–Ankle Vascular Index, before and 2 Years after Total Knee Arthroplasty in Patients with Knee Osteoarthritis

**DOI:** 10.3390/jcm12247734

**Published:** 2023-12-17

**Authors:** Yoshinori Ishii, Hideo Noguchi, Junko Sato, Ikuko Takahashi, Hana Ishii, Ryo Ishii, Kei Ishii, Kai Ishii, Shin-ichi Toyabe

**Affiliations:** 1 Ishii Orthopaedic & Rehabilitation Clinic, 1089 Shimo-Oshi, Gyoda 361-0037, Saitama, Japan; hid_166super@mac.com (H.N.); jun-sato@hotmail.co.jp (J.S.); itakahashi110@gmail.com (I.T.); 2 School of Plastic Surgery, Kanazawa Medical University, 1-1 Daigaku Uchinada, Ishikawa 920-0253, Japan; hanamed12@gmail.com; 3 Shinshu University Hospital, 3-1-1 Asahi Matsumoto, Nagano 390-8621, Japan; kmuyakyu@gmail.com; 4 Iwate Prefectural Chuo Hospital, 1-4-1 Ueda, Morioka 020-0066, Japan; kei141.0852@gmail.com; 5 Kouseiren Takaoka Hospital, 5-10 Eirakutyo Takaoka, Toyama 933-8555, Japan; kai.nd1209@live.com; 6 Niigata University Crisis Management Office, Niigata University Hospital, Niigata University Graduate School of Medical and Dental Sciences, 1 Asahimachi Dori Niigata, Niigata 951-8520, Japan; toyabe@med.niigata-u.ac.jp

**Keywords:** knee osteoarthritis, arteriosclerosis, cardio–ankle vascular index, total knee arthroplasty

## Abstract

**Purpose**: Cardiovascular disease (CVD) is a major risk factor for mortality in patients with osteoarthritis, and such comorbidities increase the risk of postoperative complications following total knee arthroplasty (TKA). Arteriosclerosis plays a major role in hemodynamic dysfunction and CVD; however, the postoperative changes in arteriosclerosis following TKA have not been evaluated. Therefore, we assessed the postoperative changes in arteriosclerosis using the cardio–ankle vascular index (CAVI) in patients undergoing TKA, and its relationships with preoperative patient characteristics. **Methods:** Arteriosclerosis was prospectively evaluated in 119 consecutive patients (140 knees) (15 males (17), 104 females (123); median age 73 years) with knee osteoarthritis who underwent TKA. CAVI was measured before and 2 years after TKA, and the relationships between CAVI and preoperative age, sex, BMI, physical activity status, comorbidities, clinical score, triglyceride concentration, cholesterol concentration, and smoking history were analyzed. **Results:** CAVI remained stable or improved in 54 joints (39%) and worsened in 86 joints (61%) 2 years post-operation. The median difference between pre- and postoperative CAVI was 0.2 (−0.3, 0.8), and the only preoperative factor associated with this change was preoperative CAVI (r = −0.469, *p* < 0.001). No other preoperative factor had a significant effect on postoperative arteriosclerosis. **Conclusions:** The results suggest that patients who undergo TKA subsequently show less severe arteriosclerosis, and the protective effect of TKA on arterial stiffness is greater in those with a higher preoperative CAVI. TKA may be an effective means of reducing the deterioration of arteriosclerosis associated with knee osteoarthritis, at least in the relatively short term.

## 1. Introduction

Patients with knee osteoarthritis experience difficulty walking owing to swelling, pain, and/or stiffness in the affected joints [1]. This leads to lower mobility, which can increase the risk of cardiovascular events [2,3] and reduce physical performance in older people, such as quadriceps muscle strength [4], balance [5,6], and gait velocity [7,8].

Total knee arthroplasty (TKA) is associated with good long-term clinical results [9,10] as a pain control measure for patients with end-stage knee osteoarthritis. Patients with osteoarthritis of the hip or knee with comorbidities, such as diabetes mellitus (DM), cancer, cardiovascular disease (CVD), or gait disturbance, are at a higher risk of death than the general population [11]. Therefore, the control of these comorbidities is very important.

The cardio–ankle vascular index (CAVI) is a marker of arterial stiffness arteriosclerosis that is based on the stiffness parameter β [12]. Measurement of CAVI is simple and has been well-standardized, and its reproducibility and accuracy are acceptable [13]. Thus, CAVI represents a promising diagnostic tool for the evaluation of arteriosclerosis [14]. There have been reports of the cessation of smoking [15] and weight loss [16] improving CAVI. However, the studies in which CAVI was used to compare the efficacy of drugs for the treatment of DM [17] and hypertriglyceridemia [18] were of short duration, ranging from 12 weeks to 6 months. To the authors’ knowledge, there have been no long-term studies regarding the relationship between the increase in physical activity associated with the reduction in pain following TKA for osteoarthritis and arteriosclerosis.

Therefore, we aimed to compare the CAVI of patients with knee osteoarthritis preoperatively and 2 years following TKA to determine how the improvement in mobility associated with pain relief affects CAVI or arteriosclerosis, and to identify preoperative factors that might affect CAVI.

## 2. Material and Methods

Written informed consent was obtained from all patients after a discussion of the protocol and possible complications. The Institutional Review Board approved the study (ID No. 10) on 10 October 2009. Of 234 patients with knee osteoarthritis who underwent TKA at our institution between October 2009 and August 2020, 119 patients (140 knees) (17 males (15), 123 females (104); median age 73 (69,78), range 55–88 years)) who agreed to undergo CAVI and for whom a complete set of preoperative and follow-up data, including CAVI, were prospectively studied. The inclusion criterion was a diagnosis of primary knee osteoarthritis. Patients who underwent revision arthroplasty, had previously undergone tibial osteotomy, had experienced tibial or femoral shaft fracture, and with rheumatoid arthritis were excluded.

The LCS^®^ Total Knee System (DePuy, Warsaw, IN, USA) was used on all patients. All TKAs were performed using a unilateral procedure under general anesthesia without using adductor canal block and femoral nerve block; all patients with bilateral disease were scheduled to undergo staged bilateral TKA. A tourniquet was used to prevent excessive intraoperative hemorrhage. One senior surgeon (Y.I.) performed all TKAs using a standardized technique with the standard medial parapatellar approach, including the necessary soft tissue releases for proper gap balancing. An anterior midline skin incision was made, extending from the level of the distal tibial tubercle to approximately 6 cm proximal to the superior border of the patella. In all knees, the femoral components were fixed without cement, and the tibial components were fixed with cement. No patellar replacement or lateral release was performed in any case. All wounds were closed in the same manner by one surgeon (Y.I.). Capsular repair was performed with an approximately 2 cm interval. A bulky compression dressing was applied.

After the first dressing change on the first postoperative day, weight bearing with a cane was permitted as tolerated under the supervision of a therapist, and additional exercises were allowed as detailed below. Passive range of motion (ROM) exercises were performed daily beginning 1 week post-operation. Patients received at least 2 h of daily physical therapy, which consisted of isometric exercises, passive ROM, active-assisted ROM, quadriceps and hamstring strengthening, and gait training, including ascending and descending stairs.

The following preoperative factors were analyzed: sex, age, height, body mass, body mass index, serum cholesterol concentration, serum triglyceride concentration, comorbidities including DM and hypertension, smoking history, American Society of Anesthesiologists (ASA) grade [19], range of knee motion, and Hospital for Special Surgery (HSS) knee score [20] (a physician-derived score). The HSS knee score is composed of seven categories: pain, function, range of motion, muscle strength, flexion deformity, instability, and subtraction. TKA that was associated with an HSS Knee Score > 90 was considered to have been successful. The clinical characteristics of the participants are summarized in Table 1.

### 2.1. Measurement of CAVI

**Figure 1:**
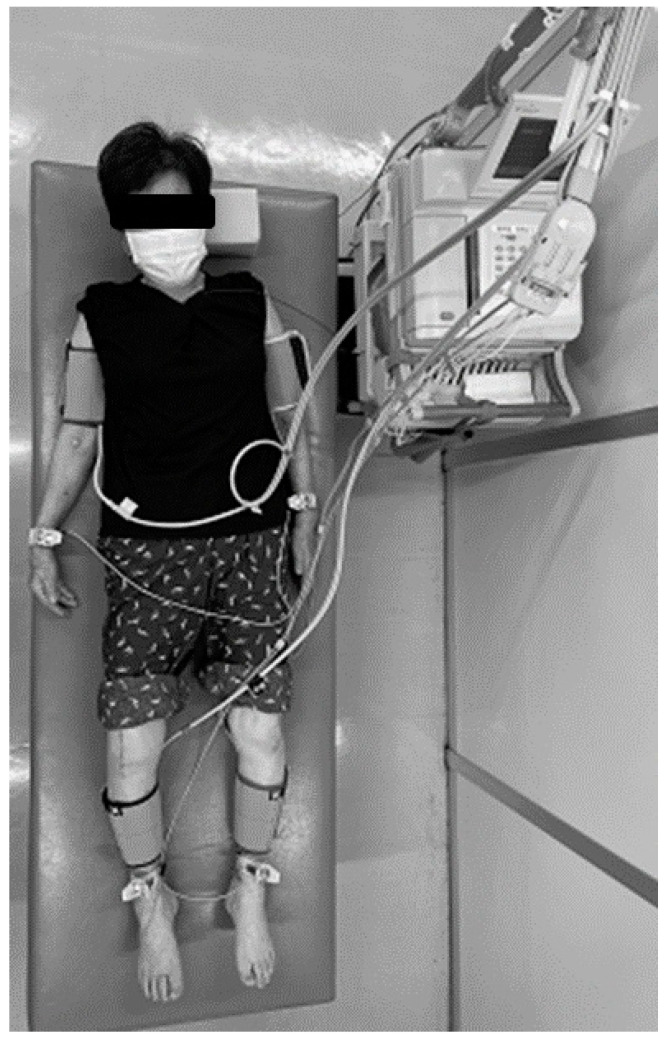
Measurement of cardio–ankle vascular index (CAVI) 2 years following a right-side total knee arthroplasty in a 77-year-old woman. First, the distance from the origin of the aorta to the ankle was measured with the patient lying supine on a bed. Next, cuffs were wrapped around the right and left upper arms and the right and left ankle joints to measure blood pressure, and a microphone that detects heart sounds was attached to the chest. The instrument automatically measured the pulse wave and blood pressure and calculated CAVI. The entire measurement took ~15 min and was painless.

CAVI was measured by a standardized method using a non-invasive blood pressure-independent device (VaSera VS-1 3000; Fukuda Denshi, Tokyo, Japan) [21] at 22–26 °C while the participants were supine. Cuffs were applied bilaterally to their upper arms and lower legs, above their ankles. Electrocardiographic electrodes and a microphone were placed on both wrists, both ankles, and sternum. An electrocardiogram, blood pressure, and waveforms for the brachial and ankle arteries were obtained (Figure 1), then pulse wave velocity (PWV) was calculated using the time between the closing sound of the aortic valve, the notch of the brachial pulse wave, and the ankle pulse wave. CAVI was calculated using this value and the following equation: CAVI = 2ρ/(systolic blood pressure − diastolic blood pressure) × (ln systolic blood pressure / diastolic blood pressure) × PWV^2^; where ρ is blood viscosity [6,21]. Based on the results of recent studies that identified significant correlations between the postoperative amelioration of pain and functional recovery up to 2 years following TKA [22,23], CAVI was measured 1 day before surgery and 2 years post-operation. The CAVI thresholds proposed by the Japan Society for Vascular Failure (<8, normal; 8–8.99, borderline; ≥9, abnormal) were used [24].

### 2.2. Reproducibility

The same investigator performed all assessments to eliminate inter-observer variability. Test–retest reliability was assessed by the same investigator in 30 participants at a 1-month interval using the intraclass correlation coefficient. The intra-class correlation coefficient was 0.788 (0.603–0.898).

### 2.3. Statistical Analysis

We estimated the sample size required to evaluate the relationships between the post-operative change in CAVI and various parameters using a power analysis prior to the study. We estimated that the sample size required to achieve a power of 0.8 with an effect size of 0.3 and alpha error of 0.05 for two-tailed Spearman’s rank coefficient was 82. Therefore, the 140 knees studied were sufficient to appropriately power the study. A post hoc power analysis revealed that the power of the study was 0.959.

Because some of the continuous datasets were not normally distributed, we used non-parametric methods for their statistical analysis, and they are expressed as median values (25th percentile, 75th percentile). The relationships between the postoperative change in CAVI and preoperative parameters were analyzed using Spearman’s rank correlation. The rank correlation coefficients were defined as strong (0.70–1.00), moderate (0.40–0.69), or weak (0.20–0.39). Unpaired continuous datasets were compared using the Mann–Whitney U test and paired continuous datasets were compared using Wilcoxon’s signed rank test. Multiple regression analysis was performed to identify independent variables that were significantly associated with the change in CAVI. A stepwise selection method was used for the significant variables. Finally, we compared the change in CAVI between participants with and without arteriosclerosis using a preoperative CAVI of 9.0 as the threshold and Pearson’s Chi-squared test. Two-tailed *p*-values of <0.05 were considered to represent statistical significance. Statistical analyses were performed using SPSS Statistics 22 (IBM, Inc., Armonk, NY, USA).

## 3. Results

**Figure 2:**
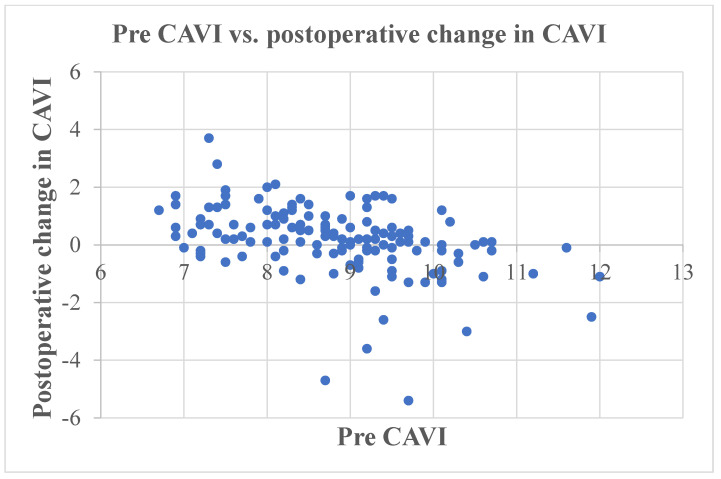
Scatter plot of the change in CAVI (postoperative − preoperative CAVI) versus preoperative CAVI. CAVI, cardio–ankle vascular index.

**Figure 3:**
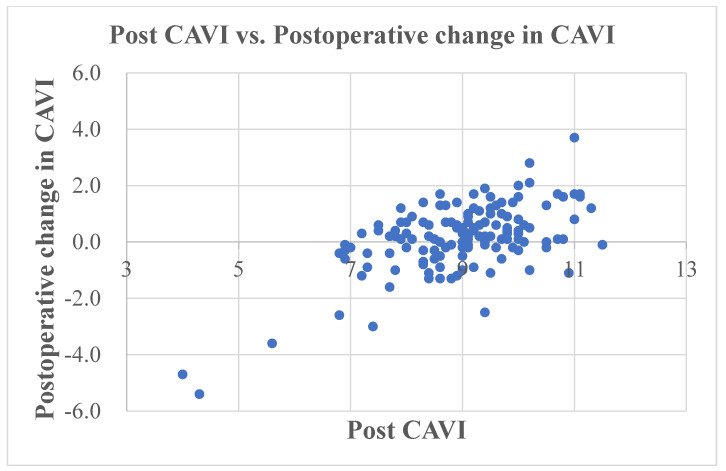
Scatter plot of the change in CAVI (postoperative − preoperative CAVI) versus postoperative CAVI. CAVI, cardio–ankle vascular index.

The preoperative CAVI was 8.8 (8.0, 9.5), and this significantly increased to 9.1 (8.3, 9.8) post-operation (*p* = 0.004). The median change in CAVI (post TKA − pre TKA) was 0.2 (−0.3, 0.8), and there was a significant negative correlation with preoperative CAVI (r = −0.469, *p* < 0.001) (Figure 2) (Table 2) and a significant positive correlation with postoperative CAVI (r = 0.525, *p* < 0.001) (Figure 3).

CAVI was maintained postoperatively in 5 joints, improved in 49 joints (39%), and worsened in 86 joints (61%) over the 2 years following the surgery (Table 3). Participants tended to show less postoperative deterioration in their arteriosclerosis when their preoperative CAVI was ≥9.0 (defined in accordance with the previously reported criterion [24]). Specifically, 28/65 (43%) of participants with arteriosclerosis and 58/75 (77%) of those without showed an improvement in or maintenance of their CAVI following TKA (*p* < 0.001 for the difference) (Table 3). Thus, the protective effect of TKA on arteriosclerosis was more marked in participants with more advanced arteriosclerosis pre-operation.

An analysis of the relationships of preoperative factors other than CAVI with the change in CAVI (Table 2 and Table 4) and comparisons of the change for participants with factors in common (Table 5) showed no significant relationships with the change in CAVI following TKA.

## 4. Discussion

We made two important findings in the present study. First, approximately 39% of the knees showed maintenance of or improvement in CAVI in the 2 years following surgery, despite previous studies having shown that CAVI worsens over time [21,25], which implies that TKA can prevent the worsening of CAVI and thus the progression of arteriosclerosis. Second, among the preoperative factors considered, only CAVI showed a *negative* correlation with the magnitude of the postoperative change. This implies that a low preoperative CAVI (corresponding to less advanced arteriosclerosis) was associated with a larger postoperative change in CAVI, whereas a higher CAVI (corresponding to more advanced arteriosclerosis) was associated with a smaller change in CAVI. Thus, when a patient has a low CAVI, TKA does not tend to prevent an increase in arterial stiffness; by contrast, when a patient has a higher preoperative CAVI, TKA offers greater protection against a worsening of arterial stiffness during the 2 years following TKA. These findings are consistent with the results of the comparison of the prevalence of postoperative maintenance of or improvement rate in CAVI between participants with and without arteriosclerosis, using a preoperative CAVI threshold of 9.0. Furthermore, the prevalence of maintenance of or improvement in CAVI was significantly higher in participants with arteriosclerosis and a high preoperative CAVI than in those without arteriosclerosis and a lower CAVI.

There have been reports of the progression of arteriosclerosis and an associated worsening of CVD owing to the lower physical activity associated with pain in patients with osteoarthritis [2,3]. In addition, it has been reported that the mortality rate of older patients with complications of osteoarthritis is higher than that of those without [11]. Osteoarthritis treatment in older individuals [11] and increasing their physical activity level have been advocated as preventive measures [2]. However, to the authors’ knowledge, there have been no long-term studies of the effects of changes in physical activity before and after TKA on the cardiovascular system. In the present study, it can be assumed that there was an increase in physical activity because the clinical knee scores [19] of the participants, including their pain and walking ability ratings, markedly improved following TKA. We found that TKA was associated with the maintenance of or improvement in CAVI in approximately 39% of the participants over 2 years.

There have been two previous studies of healthy individuals showing that CAVI worsened by 0.5 over 10 years [21] and 0.47 ± 0.68 over 5 years [25]. Therefore, we can assume that CAVI would have worsened by approximately 0.1–0.2 over 2 years in the present healthy participants. When the amount of exercise that patients with end-stage osteoarthritis can do is restricted, as in the older patients in the present study, it is not unreasonable to assume that they were likely to show more deterioration in their CAVI than the participants in the two previous studies. Thus, TKA for patients with painful end-stage knee osteoarthritis may be regarded as a surgical means of slowing the progression of age-related arteriosclerosis that is as effective as non-surgical treatments, such as the cessation of smoking [15], weight loss [16], and medication [17,18]. In the future, a combination of non-surgical and surgical approaches may be expected to become treatment options for patients with end-stage osteoarthritis and concomitant arteriosclerosis.

Of the factors considered, only preoperative CAVI was found to *negatively* correlate with the change in CAVI following TKA. There may be two possible explanations for this finding. First, it is possible that in patients with advanced arteriosclerosis and a high preoperative CAVI, there might be potential for greater improvements in postoperative CAVI because of greater blood flow during exercise. The beneficial effects of an increase in physical activity have been reported to be underpinned by an increase in blood flow, leading to greater shear stress, stimulating the production of endothelium-derived nitric oxide, which induces structural adaptation of the arteries [26]. Although we did not measure the number of steps taken by the participants, it is reasonable to assume that there was an increase in exercise, judging from the marked improvement in HSS score [20], which included an assessment of walking ability, following TKA. In addition, in participants with borderline or no arteriosclerosis and a low preoperative CAVI, there may be less room for improvement in CAVI because their arteries are less stiff, and the positive effect of greater blood flow may not be reflected in further improvement in postoperative CAVI.

The findings of Tabara et al. [25] are consistent with the present findings. They performed a large longitudinal study in a sample of the general population that showed an inverse association between CAVI at baseline and the change in CAVI (CAVI 5 years after baseline − baseline CAVI). The explanation given for this inverse association was that there was a capacity for large arteries to stiffen in individuals with a low CAVI at baseline, and a ‘regression to the mean’ statistical artifact: if CAVI was high or low at the first measurement, it would tend to be closer to the mean value at the second measurement in the absence of an intervention. On this basis, we supposed that although a positive effect of TKA on CAVI would be expected, owing to the increase in blood flow stimulated by exercise, a negative effect owing to deterioration over time would also occur simultaneously, such that CAVI would improve when the positive effect predominated and worsen when the negative effect predominated.

There have been previous reports of associations of patient factors with the worsening of CAVI over time, including age [21,25], male sex [25,27,28,29], obesity [17,30], DM [17,27,28], high triglycerides [18,29,31], high cholesterol [28,29], and smoking [25,27,32]. In addition, stopping smoking [15] and weight loss [16] have been reported to be associated with improvements in CAVI, and CAVI has also been used to compare the efficacy of agents targeting DM [17] and hyperlipidemia [18]. However, all of the previous studies were of short duration, ranging from 12 weeks to 6 months. The preoperative factors considered in the present study included all these factors, but none were found to be associated with the magnitude of the postoperative change in CAVI. This may be, at least in part, because all of the participants in the present study had ASA grades I or II, which may not have been severe enough to significantly affect the results. Therefore, we plan to study patients with ASA grade III in the future to increase the validity of the present findings.

There were a number of limitations to the present study. First and foremost, there was no comparator group of patients that did not undergo TKA. However, while such a control group may have been necessary to more accurately judge the effects of TKA on CAVI, it is not clinically realistic to follow patients with knee pain and dysfunction severe enough to require surgery over the long term. Second, the present study was performed at a single institution, in participants of a single ethnicity, and predominantly in women. Racial specificity has been reported for arteriosclerosis [33,34,35]; therefore, multi-center studies in multiple countries would be desirable to examine the validity of the present findings. Third, laboratory data were not available from the 2-year follow-up examination; therefore, comparisons with the preoperative period could not be made. We plan to rectify this in future studies. Fourth, the follow-up period was relatively short; a medium-to-long follow-up period of 5–10 years would be essential to fully characterize the effects of TKA on CAVI alongside the effects of aging. These might be significant limitations that affect the validity and generalizability of the study’s findings. Finally, the number of participants who were followed up 2 years post-operation was relatively low, at approximately 60%. A likely explanation for this is a reduction in the number of patient visits for regular check-ups because of the COVID-19 pandemic. We hope that the next follow-up survey (after 5–10 years) will have a higher participation rate than the 2-year survey. However, despite these limitations, this is the first report to demonstrate that TKA may be an effective means of reducing the deterioration of arteriosclerosis associated with knee osteoarthritis, at least in the relatively short term.

## 5. Conclusions

We found that TKA for the treatment of knee osteoarthritis prevented age-related deterioration in arteriosclerosis in ~39% of patients. This implies that TKA can be expected to slow the progression of arteriosclerosis. This effect was found to be more marked in patients with a higher preoperative CAVI. Medium- and long-term follow-up studies of CAVI following TKA surgery should be performed to validate these findings.

## Figures and Tables

**Table 1:** Characteristics of the participants.

Parameter (patients/knees)	N = 119/140
Sex (Male (knees)/Female (knees))	15 (17)/104 (123)
Height (cm)	150 (145, 155)
Body mass (kg)	58 (52, 66)
Body mass index (kg/m^2^)	26 (24, 28)
Age (years)	73 (69, 79)
Smoking history (yes/no) number of knees	6/134
Diabetes mellitus (yes/no) number of knees	19/121
Hypertension (yes/no)	93/47
Preoperative serum cholesterol concentration (nmol/L)	5.2 (4.8, 5.9)
Preoperative serum triglyceride concentration (nmol/L)	1.5 (1.1, 2.0)
Preoperative range of knee motion (°)	100 (90, 120)
Postoperative range of knee motion (°)	110 (100, 120)
ASA Grade	I 20, II 120
Preoperative HSS	44 (35, 51)
Postoperative HSS	92 (92, 94)
Preoperative CAVI	8.8 (8.0, 9.5)
Postoperative CAVI	9.1 (8.3, 9.8)

Data are presented as n or median (25th percentile, 75th percentile). ASA, American Society of Anesthesiologists [19]; HSS, Hospital for Special Surgery [20]; CAVI, cardio–ankle vascular index.

**Table 2:** Relationships of the change in CAVI with preoperative study variables, according to Spearman’s rank correlation coefficient.

Variable	r	*p*
**Pre CAVI**	**−0.469**	**<0.001**
Age		0.652
Height		0.278
Body mass		0.744
Body mass index		0.367
HSS score		0.246
ROM	0.175	0.039
Cholesterol		0.306
Triglycerides		0.713

Values in bold are statistically significant. CAVI, cardio–ankle vascular index; HSS, Hospital for Special Surgery [20]; ROM, range of motion; Pre, preoperative.

**Table 3:** Comparison of the change in CAVI between participants with or without arteriosclerosis according to their preoperative CAVI, using 9.0 as the threshold and Pearson’s Chi-squared test.

Preoperative Arteriosclerosis	CAVI < 9.0 (No Arteriosclerosis)	CAVI ≥ 9.0 (Arteriosclerosis)	Total
Maintained or improved	17 (23%)	37 (57%)	54 (39%)
Deteriorated	58 (77%)	28 (43%)	86 (61%)
*p* < 0.001	75	65	140

Data are numbers of knees (percentage). CAVI, cardio–ankle vascular index.

**Table 4:** Results of the multiple regression analysis using a stepwise variable selection method.

Parameter	B	S.E.	Β	Sig.	95% CI	
					Lower	Upper
**Preoperative CAVI**	−0.478	0.085	−0.432	<0.001	−0.646	−0.310
**Constant**	4.368	0.753		<0.001	2.879	5.857

S.E., standard error; Sig., significance; CI, confidence interval; CAVI, cardio–ankle vascular index.

**Table 5:** Comparisons of the change for participants with factors in common.

Variable	Change in CAVI Following TKA in Each Subgroup		*p* Value
Sex	Male (17)	Female (123)	
	0.2 (−0.2, 0.8)	0.2 (−0.3, 0.8)	0.848
ASA	I (20)	II (120)	
	0.2 (−0.3, 0.7)	0.2 (−0.2, 0.8)	0.383
Smoking	Yes (6)	No (134)	
	0.1 (−0.5, 0.7)	0.2 (−0.3, 0.8)	0.248
Hypertension	Yes (93)	No (47)	
	0.2 (−0.2, 0.8)	0.3 (−0.4, 0.8)	0.788
Diabetes mellitus	Yes (19)	No (121)	
	0.3 (−0.1, 1.0)	0.2 (−0.4, 0.7)	0.469

Data are presented as number of knees and were compared using the Mann–Whitney U test. CAVI, cardio–ankle vascular index; ASA, American Society of Anesthesiologists [19], TKA, total knee arthroplasty.

## Data Availability

The datasets used and/or analyzed during the current study are available from the corresponding author upon reasonable request.
